# Inflammatory myofibroblastic tumor of the prostate after transurethral resection of the prostate with negative expression of anaplastic lymphoma kinase: a case report

**DOI:** 10.1590/1516-3180.2017.0079070417

**Published:** 2018-06-04

**Authors:** Jie Zeng, Rong-Quan He, Wei-Guang Mo, Zhi-Gang Peng, Jie Ma, Jin-Cai Zhong, Chao-Hua Mo, Mei-Jiao Qin, Xiao-Hua Hu

**Affiliations:** I MD, MSc. Postgraduate Student, Department of Medical Oncology, First Affiliated Hospital of Guangxi Medical University, Nanning, Guangxi, China.; II MD. Postgraduate Student, Department of Medical Oncology, First Affiliated Hospital of Guangxi Medical University, Nanning, Guangxi, China.; III MSc. Technician, Department of Pathology, First Affiliated Hospital of Guangxi Medical University, Nanning, Guangxi, China.; IV MD, MSc. Professor, Department of Medical Oncology, First Affiliated Hospital of Guangxi Medical University, Nanning, Guangxi, China.; V MD, MSc. Professor, Department of Medical Oncology, First Affiliated Hospital of Guangxi Medical University, Nanning, Guangxi, China.; VI MD, MSc. Professor, Department of Medical Oncology, First Affiliated Hospital of Guangxi Medical University, Nanning, Guangxi, China.; VII MD, MSc. Attending Physician and Postgraduate Student, Department of Pathology, First Affiliated Hospital of Guangxi Medical University, Nanning, Guangxi, China.; VIII MD, MSc. Postgraduate Student, Department of Medical Oncology, First Affiliated Hospital of Guangxi Medical University, Nanning, Guangxi, China.; IX MD, MSc. Professor, Department of Medical Oncology, First Affiliated Hospital of Guangxi Medical University, Nanning, Guangxi, China.

**Keywords:** Granuloma, plasma cell., Inflammation., Prostate.

## Abstract

**CONTEXT:**

Inflammatory myofibroblastic tumors are a rare type of soft-tissue tumor. Inflammatory myofibroblastic tumors are characterized by rearrangements involving the anaplastic lymphoma kinase gene locus on 2p23.

**CASE REPORT:**

We report the case of a 67-year-old Chinese male who presented with dysuria and fever. Magnetic resonance imaging showed an irregular prostatic mass with an isointense signal and obscure boundary. Histopathological evaluation showed that the mass consisted mainly of spindle-shaped cells. Immunohistochemical evaluation showed that the tumor cells were negative for anaplastic lymphoma kinase.

**CONCLUSIONS:**

Inflammatory myofibroblastic prostate tumors are rare lesions with unclear etiology. The pathological diagnosis is very important.

## INTRODUCTION

Inflammatory myofibroblastic tumors can be found in various parts of the body and are frequently identified in the lung or abdominal cavity of children and young adults. Inflammatory myofibroblastic tumors of the urinary tract present more often in kidneys. Prostatic inflammatory myofibroblastic tumors are extremely rare. Since the first case in 2012, only one further case of prostatic inflammatory myofibroblastic tumor has been reported in the literature.^1^,^2^ The usual clinical presentation of inflammatory myofibroblastic prostate tumors consists of dysuria and acute urinary retention. Differentiation of inflammatory myofibroblastic prostate tumors from malignant prostate tumors through imaging and laboratory tests is difficult. A case of prostatic inflammatory myofibroblastic tumor observed after transurethral resection of the prostate to treat prostate hyperplasia in a 67-year-old man is presented in this report.

## CASE REPORT

A 67-year-old Chinese male presented with dysuria and fever for a month. He had a history of transurethral resection of the prostate to treat prostate hyperplasia four years earlier. Laboratory tests such as blood count and urinalysis were within the normal range. His serum total prostate-specific antigen level was 8.49 ng/ml (normal reference range: 0.00-4.00 ng/ml) and his free prostate-specific antigen level was 1.42 ng/ml (normal reference range: 0.00-0.93 ng/ml).

**Figure 1.**
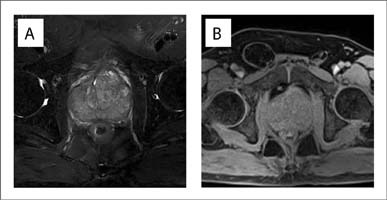
Irregular prostatic mass showing an isointense signal on T1-weighted images (A) and T2-weighted images (B), with an obscure boundary.

**Figure 2.**
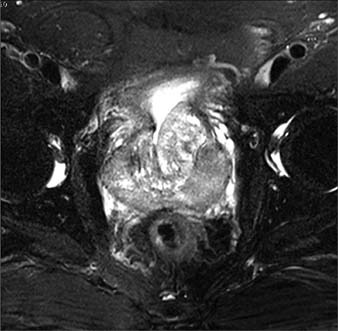
Contrast-enhanced magnetic resonance imaging showing clear enhancement in the early phase of the lesion.

Subsequently, multiple echoless masses and calcification were found using ultrasonography. Magnetic resonance imaging then demonstrated an irregular prostatic mass with an obscure boundary and isointense signal on T1 and T2-weighted images (Figure 1A and Figure 1B). The lesion showed obvious enhancement in the early phase, on a contrast-enhanced magnetic resonance imaging scan (Figure 2). Multiple enlarged lymph nodes were found in the right iliac vascular area and in the inguinal area bilaterally.

**Figure 3.**
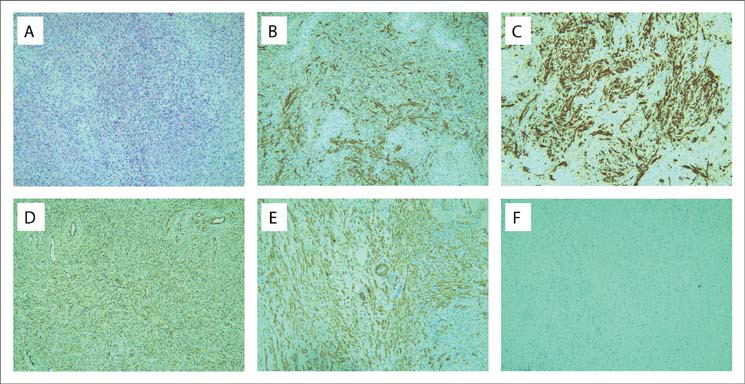
Histopathological and immunohistochemical sections through a prostatic inflammatory myofibroblastic tumor. The tumor consisted mainly of spindle-shaped cells and a chronic inflammatory component consisting of plasma cells (A); hematoxylin-eosin staining (×100). Immunohistochemical sections showed that the tumor cells were positive for desmin (B), calponin (C), vimentin (D) and smooth muscle actin (SMA) (E), but were negative for anaplastic lymphoma kinase (ALK) (F).

A transperineal biopsy of the prostate was performed on this lesion because the diagnosis was still unclear. Histopathological evaluation showed that the mass consisted mainly of spindle-shaped cells and a chronic inflammatory component consisting of plasma cells (Figure 3A). Immunohistochemical evaluation showed that the tumor cells were positive for myoepithelial markers, including desmin, calponin, vimentin, actin and smooth muscle actin (Figure 3B-3E), but that they were negative for CD117, CD10, S100 and anaplastic lymphoma kinase (Figure 3F).

Based on the histological and immunohistochemical findings, a pathological diagnosis of inflammatory myofibroblastic tumor was made. No further specific treatment was provided. The patient was followed up for two years and no evidence of recurrence or metastasis was noted.

## DISCUSSION

Inflammatory myofibroblastic tumors were once considered to be inflammatory pseudotumors, xanthogranulomas, plasma-cell granulomas or plasma-cell pseudotumors.^3^ However, inflammatory myofibroblastic tumors and inflammatory pseudotumors are completely different pathological concepts with different pathological manifestations. Inflammatory pseudotumors are characterized by an inflammatory infiltrate consisting of lymphocytes, plasma cells and histiocytes admixed with variable proportions of fibroblasts and myofibroblasts. On the other hand, inflammatory myofibroblastic tumors were classified as soft-tissue tumors by the World Health Organization in 2002, and consist mainly of differentiated myofibroblastic spindle cells, usually accompanied by numerous plasma cells, eosinophilic granulocytes and/or lymphocytes. Inflammatory myofibroblastic tumors are characterized by rearrangements involving the anaplastic lymphoma kinase gene locus on 2p23.^4^

The etiology of inflammatory myofibroblastic tumors remains unknown.^5^ Inflammatory myofibroblastic tumors are frequently associated with inflammation, surgery and trauma. Therefore, inflammatory myofibroblastic tumors may be the body’s response to injury caused by hyperplasia.

The neoplastic processes of inflammatory myofibroblastic tumors are characterized by gene rearrangement or fusion. Next-generation sequencing revealed that 85% of the cases evaluated harbored kinase fusions involving anaplastic lymphoma kinase, ROS1 or PDGFβ. The fusion partners identified thus far include the TPM3/4, CLTC and RANBP2 genes. A subset of anaplastic lymphoma kinase-negative inflammatory myofibroblastic tumors demonstrated ROS-1 gene fusions.^4^,^6^

Inflammatory myofibroblastic tumors can be found primarily in children and young adults, and they rarely occur in patients over 40 years old.^7^ Although inflammatory myofibroblastic tumors are more commonly recognized in the lungs, they can also be observed in several other sites including the breasts, liver, spleen, thyroid, pancreas, urinary tract, peritoneum, retroperitoneum, lymph nodes, gastrointestinal tract and central nervous system.^8^ Inflammatory myofibroblastic tumors of the urinary tract have been reported more often in the kidneys.

Prostatic inflammatory myofibroblastic tumors are extremely rare. Since the first case in 2012, only one further case of inflammatory myofibroblastic prostate tumor has been reported (Table 1).^1^,^2^ In the present case, we reported that an inflammatory myofibroblastic tumor was found in an elderly man after transurethral resection of the prostate to treat prostate hyperplasia. This case was negative for anaplastic lymphoma kinase.

**Table 1.** Search of the literature in medical databases for inflammatory myofibroblastic tumor of the prostate. The literature search was conducted on July 28, 2017

**Database**	**Search strategies**	**Papers found**	**Related papers**
MEDLINE (via PubMed)	#1 (“Granuloma, Plasma Cell”[Mesh]) #2 (Prostate”[Mesh]) #3 (“Transurethral Resection of Prostate”[Mesh]) #4 #2 OR #3 #5 #1 AND #4	3	2
Embase (via Elsevier)	#1(inflammatory myofibroblastic tumor) OR (inflammatory myofibroblastic tumour) OR (Granuloma Plasma Cell) #2 Prostate OR (Transurethral Resection of Prostate) #3 #1 AND #2	17	2
LILACS (via BVS)	#1 mh:(Granuloma, Plasma Cell) #2 mh:(Prostate) #3 mh:(Transurethral Resection of Prostate) #4 #2 OR #3 #5 #1 AND #4	2	1

BVS = Biblioteca Virtual em Saúde; LILACS = Literatura Latino Americana e do Caribe em Ciências da Saúde.

The final diagnosis of an inflammatory myofibroblastic tumor cannot be made from the features of the clinical manifestation, laboratory tests or radiological examination. The clinical manifestations of inflammatory myofibroblastic tumors are diverse in different sites. Urinary inflammatory myofibroblastic tumors present with obstructive symptoms such as dysuria, urinary frequency and urinary retention. Rectal examination may reveal a palpable mass. Inflammatory myofibroblastic tumors usually demonstrate low signal intensity on both T1 and T2-weighted images and show obvious enhancement on contrast-enhanced magnetic resonance imaging scans.^9^

However, these features are not distinctly different from those of prostate hyperplasia and prostate cancer. Therefore, the pathological diagnosis is especially important. These tumors are infiltrated by plasma cells, lymphocytic plasma cells, eosinophils and other inflammatory cells. Immunohistochemically, the spindle cells are positive for expression of muscle-derived proteins. Vimentin is often expressed diffusely, whereas smooth muscle actin and desmin are expressed focally or diffusely.^10^

This patient showed symptoms of dysuria, with abnormal prostate-specific antigen levels. A definite diagnosis cannot be made from symptoms, laboratory testing or imaging examinations. Ultimately, the diagnosis of an inflammatory myofibroblastic tumor was made using histological and immunohistochemical evaluations.

A variety of treatment methods for inflammatory myofibroblastic tumors have been reported, including chemotherapy, radiation therapy, non-steroidal anti-inflammatory drugs and glucocorticoid therapy. Additionally, there is no exact index to predict the risk of recurrence and metastasis.

Responses to the tyrosine kinase inhibitor crizotinib have been documented in patients with anaplastic lymphoma kinase-positive inflammatory myofibroblastic tumors. However, a new study by Lovly et al. found that this also had clear curative effects in patients with ROS1 fusion.^6^ For extrapulmonary inflammatory myofibroblastic tumors, surgical excision is still preferred.^11^,^12^ Coffin et al. reported that negative expression of anaplastic lymphoma kinase correlated with a risk of recurrence and metastasis.^6^,^13^,^14^ However, a review of extrapulmonary inflammatory myofibroblastic tumors documented a recurrence rate of 31% among anaplastic lymphoma kinase-negative tumors and 69% among anaplastic lymphoma kinase-positive tumors. Anaplastic lymphoma kinase gene rearrangements occurred in approximately 50-75% of cases of inflammatory myofibroblastic tumors.^7^,^15^

Neither of the previous reports of prostatic inflammatory myofibroblastic tumors measured anaplastic lymphoma kinase levels. One of these patients suffered from recurrence and distant metastasis and subsequently died.^1^,^2^

The patient of our report did not express anaplastic lymphoma kinase. No further specific treatment was provided. The patient was followed up for two years and no evidence of recurrence or metastasis was noted. This suggests that treatment approaches for inflammatory myofibroblastic tumor patients can vary and surgery may not be essential.

## CONCLUSION

In conclusion, prostatic inflammatory myofibroblastic tumors are rare lesions with unclear etiology. A definitive diagnosis cannot be made from symptoms, laboratory testing and imaging studies. The pathological diagnosis is very important. Regarding treatment methods, although surgery is not essential for treating prostatic inflammatory myofibroblastic tumors that are negative for anaplastic lymphoma kinase expression, long-term follow-up is necessary.
